# Topographic determinants of foot and mouth disease transmission in the UK 2001 epidemic

**DOI:** 10.1186/1746-6148-2-3

**Published:** 2006-01-16

**Authors:** Nicholas J Savill, Darren J Shaw, Rob Deardon, Michael J Tildesley, Matthew J Keeling, Mark EJ Woolhouse, Stephen P Brooks, Bryan T Grenfell

**Affiliations:** 1 Statistical Laboratory, Centre for Mathematical Sciences, University of Cambridge, Wilberforce Road, Cambridge, CB3 0WB, UK; 2 Institute of Infection and Immunology Research, School of Biological Sciences, University of Edinburgh, Kings Buildings, West Mains Road, Edinburgh, EH9 3JT, UK; 3 Veterinary Clinical Studies, R(D)SVS, University of Edinburgh, Easter Bush Veterinary Centre, Roslin, Midlothian, EH25 9RG, UK; 4 Ecology and Epidemiology Group, Department of Biological Sciences, University of Warwick, Gibbet Hill Road, Coventry, CV4 7AL, UK; 5 Center for Infectious Disease Dynamics, Department of Entomology and Biology, Pennsylvania State University, University Park, PA 16802, USA; 6 Fogarty International Center, National Institutes of Health, Bethesda, MD 20892, USA; 7 Cambridge Infectious Diseases Consortium, Department of Veterinary Medicine, University of Cambridge, Madingley Road, Cambridge, CB3 0ES

## Abstract

**Background:**

A key challenge for modelling infectious disease dynamics is to understand the spatial spread of infection in real landscapes. This ideally requires a parallel record of spatial epidemic spread and a detailed map of susceptible host density along with relevant transport links and geographical features.

**Results:**

Here we analyse the most detailed such data to date arising from the UK 2001 foot and mouth epidemic. We show that Euclidean distance between infectious and susceptible premises is a better predictor of transmission risk than shortest and quickest routes via road, except where major geographical features intervene.

**Conclusion:**

Thus, a simple spatial transmission kernel based on Euclidean distance suffices in most regions, probably reflecting the multiplicity of transmission routes during the epidemic.

## Background

The UK 2001 epidemic of foot and mouth disease highlighted the need for national governments to have well thought out and workable contingency plans to control the spread of highly infectious animal diseases. These plans must be based on quantitative predictions of epidemic size and extent under various conditions which, in turn, must be based on an understanding of how disease spreads between livestock premises. For a disease like foot and mouth that has a multitude of transmission routes between premises, predicting the course of an epidemic is complicated by demographic and topographic heterogeneity. The UK 2001 foot and mouth epidemic has furnished us with unique data on the temporal and spatial spread of this disease. This, coupled with demographic data of livestock holdings within the UK, gives us the opportunity to study in detail the various risk factors associated with disease spread. In this paper we focus on the effect caused by geographical features within the UK landscape.

**Figure 1:**
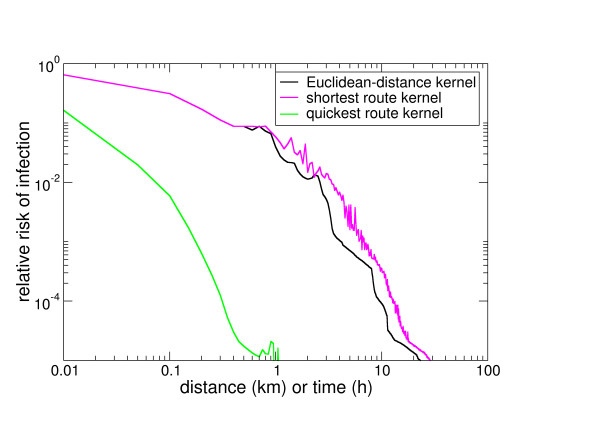
Euclidean-distance based kernel (black line) estimated from DEFRA contact tracing after 23rd February 2001. Shortest route (magenta line) and quickest route (green line) kernels estimated from the Euclidean-distance based kernel and the Devon demographic data.

Foot and mouth disease is a viral infection of mainly domesticated and wild cloven-hoofed animals. It is an acute, febrile disease typically associated with lesions on the feet and in the mouth [1]. There are seven serotypes of foot and mouth disease virus (FMDV) which may differ in their transmission characteristics [2]. The introduction of type O FMDV into the UK in early 2001 resulted in reported infection in livestock on 2,026 mainland premises. 4.2 million animals on over 10,000 premises were slaughtered in the ensuing operation to wipe out the disease [2]. Before the nationwide movement ban on 23rd February 2001, the disease was seeded across the UK by the transportation of infected sheep. After the ban, disease spread became more highly localised – about 50% of infections occurred within 3 km of an infectious infected premises (IP), and about 80% occurred within 10 km [3-5]. Early models of the epidemic quantified the risk of transmission using a simple concept called a transmission kernel [6]. The kernel encapsulated multiple and diverse transmission routes in a simple function of Euclidean distance between infectious and susceptible farms. An estimate of the kernel, based on the UK Government's Department of Environment, Food and Rural Affairs (DEFRA) contact tracing data, is shown as the black line in Fig. 1[3,5].

Using Euclidean distance between farms in the transmission kernel was a first approximation to quantify the effect of spatial separation on FMDV transmission. However, contact tracing of infection by DEFRA highlighted several important transmission routes that occurred via road after the movement ban. These included movement of vehicles, personnel, milk tankers, farm equipment and livestock [7]. Thus, road-based measures may be better predictors of transmission risk than just simple Euclidean distance, especially in areas where roads go around large geographical features such as hills, rivers and estuaries. Showing this, however, is complicated by the non-unique, non-linear relationships between Euclidean distance and road-based measures, and by the fact that Euclidean distance is already a risk factor. Nevertheless, with detailed descriptions of the UK road network and the UK 2001 foot and mouth disease dynamics, we have developed a statistical test that can detect risk associated with road-based measures. In this paper we consider shortest and quickest routes.

Briefly, the test looks for a significant difference in the mean shortest or quickest route between farms where a possible transmission occurred and the mean shortest or quickest route between farms where no transmission occurred; the rational being that if shortest or quickest routes are risk factors, farms closer to an IP by road are more likely to have been infected than farms farther away. A detailed description of the test is given in Methods

## Results and Discussion

We analyse the regional epidemics in Devon, Cumbria, Dumfries and Galloway, Settle and the Welsh borders. Counties included in the analyses are given in Table 1.

**Figure 2:**
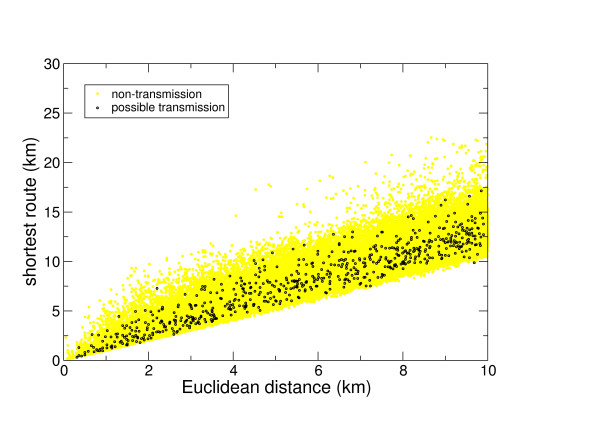
Euclidean versus shortest route for non-transmissions (yellow closed circles), and possible transmissions (black open circles) for the Devon epidemic after 23rd February 2001.

Shortest route versus Euclidean distance for the epidemic in Devon for all possible transmission events after 23rd February and within 10 km are shown as open black circles in Fig. 2. All non-transmissions in Devon after 23rd February and within 10 km are shown as yellow closed-circles.

The *p*-value for the null hypothesis that the difference in the mean shortest route between possible transmissions and the mean shortest route between non-transmissions could have arisen by chance is 0.55 (Table 2). Therefore we conclude that shortest route in Devon is no better a predictor of risk than Euclidean distance. Quickest route is similarly no better: *p = *0.91. These conclusions are robust to assumptions about latent period and species transmissibilities (Table 2). Similar conclusions can be drawn for the other epidemics in the Welsh borders, Cumbria, Settle and Dumfries and Galloway. When the analysis is done with only those 65% of IPs that were positively confirmed as infected by the Institute of Animal Health at Pirbright, our conclusions remain unchanged (see Table 3).

These tests show that shortest and quickest routes are no better predictors of transmission risk than Euclidean distance. However, it does not prove that they are any worse. In order to test this we turn the analysis around and ask if Euclidean distance is a better predictor of transmission risk than shortest or quickest route. This requires the calculation of shortest and quickest route transmission kernels (see Methods and Fig. 1). The *p*-values for the null-hypothesis that the difference in the mean Euclidean distance between possible transmissions and the mean Euclidean distance between non-transmissions could have arisen by chance are given in Table 2. For all regions and all parameter values the *p*-values are significant, strongly suggesting that Euclidean distance is a better predictor of risk than shortest and quickest routes. When the analysis is done with only those IPs that were positively confirmed as infected the results are not significant for shortest route in Devon, Dumfries and Galloway and the Welsh Borders, and for quickest route in the Welsh Borders. This is most likely due to small sample sizes reducing the power of the test (see *n *in Table 3).

**Figure 3:**
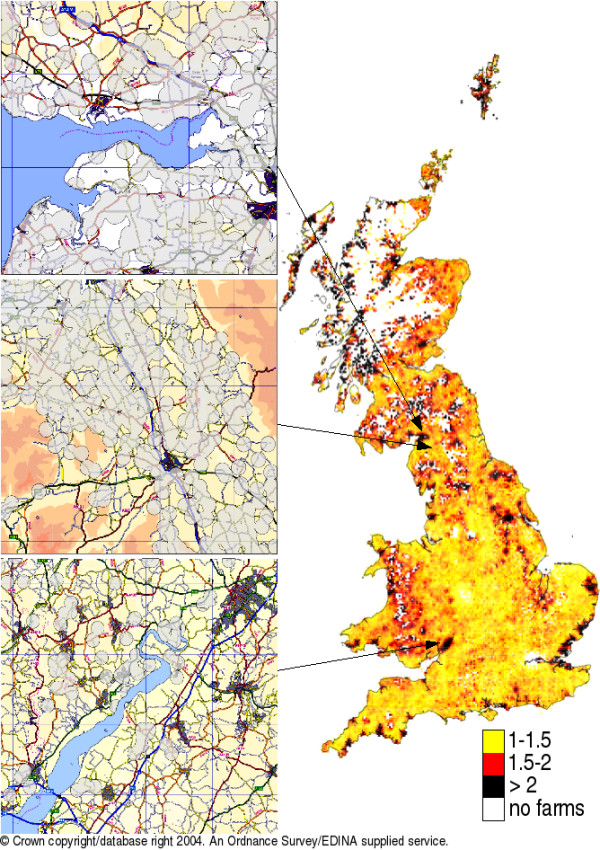
UK map of average ratio of shortest route to Euclidean distance for all pairs of livestock farms within 10 km of each other. Averages are taken over 3 × 3 km^2 ^areas. Top inset: Solway Firth, middle inset: M6 Motorway in Cumbria, bottom inset: river Severn in Gloucestershire showing the M4/M48 and A40 roads that cross it. Grid lines on insets are spaced every 10 km. Light grey areas show infected areas.

The above statistical test acts on the regional scale, therefore, distance-based risk associated with specific geographical features is lost when we analyse over a region with widely variable topography. Of particular interest are geographical features that act as barriers to direct FMDV transmission between farms. Such features include lakes, rivers, estuaries, hills, mountains, railway lines and major arterial roads. A key question is whether transmission across such features is better modelled using Euclidean-distance or some other distance measure; road-based measures being the most obvious candidates because the road network is shaped by such geographical features: roads go around lakes; are diverted, sometimes by long distances, by rivers, estuaries, railway lines and motorways; and tend to follow valleys rather than climb hills and mountains. This is demonstrated in Fig. 3. Mean shortest route between pairs of low-lying, inland farms is usually less than about 50% longer than Euclidean distance (yellow). Areas where mean shortest route is greater than 50% longer than Euclidean distance (red and black) include many rivers and estuaries, the East Anglian Fens, the Norfolk Broads, moorland, and hill and mountain ranges. Of particular interest in terms of the UK 2001 epidemic are the Solway Firth (Fig. 3, top inset) and the river Severn (Fig. 3, bottom inset) where infected premises were observed at the same time during the epidemic on both sides of these estuaries. In the absence of wind-borne viral plumes [8], transmission did not occur directly over these large bodies of water. Models using a Euclidean-distance based transmission kernel may over-estimate the number of transmission events across these features; therefore a shortest route based kernel may be more appropriate. In Methods we describe a statistical test that detects if transmission between farms on opposite sides of a barrier is better modelled by a shortest route based kernel or a Euclidean-distance based one. The Solway Firth estuary partially separates Cumbria from Dumfries and Galloway. Many farms were infected on both sides of the Solway Firth throughout March and April. Because it is so broad, any transmission between farms on its opposite sides most probably occurred via road. The *p*-value for the test is less than 0.001 (*n *= 2429); a highly significant result suggesting, not unexpectedly, that transmission between farms on opposite sides of the Solway Firth is best modelled using a shortest route based transmission kernel.

The river Severn and its estuary are crossed by the M4/M48 Motorway in the southwest and by the A40 trunk road in the northeast, which themselves are about 40 km apart (Fig. 3, bottom inset). The *p*-value for the test is less than 0.001 (*n *= 672); a highly significant result suggesting that transmission between farms on opposite sides of the Severn is best modelled using a shortest route based transmission kernel.

**Figure 4:**
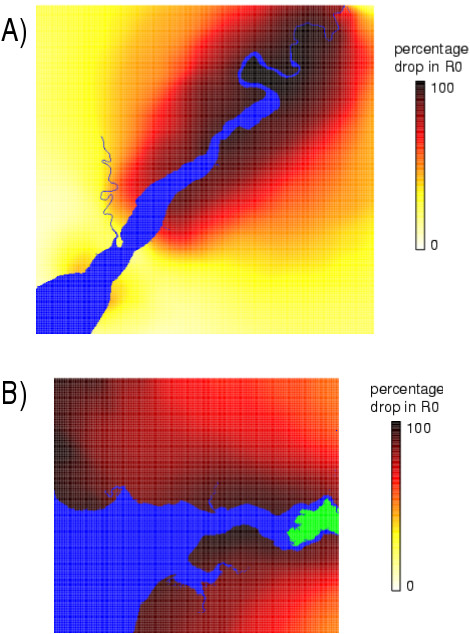
Percentage reduction in *R*_0 _across the river Severn (A) and the Solway Firth (B) due to their presence. For every farm *i*, we calculate 1 - ∑_*j *_*S*_*j*_*K*(*r*_*i*,*j*_)/∑_*j *_*S*_*j*_*K*(e_*i*,*j*_), where *j *is in the set of all farms within 10 km of *i *and on the opposite side of the estuary to it, *S*_*j *_is the susceptibility of farm *j *given by *S*_*j *_= *N*_*s*,*j *_+ 10*N*_*c*,*j *_[5] and *e*_*i*,*j *_is Euclidean distance and *r*_*i*,*j *_is shortest route. These values are Gaussian smoothed over a 5 km radius and then averaged. Blue: water, green: no farms.

In Fig. 4A we plot the percentage reduction in the case reproduction ratio (*R*_0_) across the river Severn due to its presence. Close to the river and midway between the A40 and M4 bridge crossings, the reduction in *R*_0 _is close to 100%: farms in this area are therefore unlikely to infect farms on the opposite side of the river. As we move further away from the river or toward the bridges the reduction in *R*_0 _is less severe, as one would expect. A similar conclusion naturally applies to the Solway Firth (Fig. 4B).

We have also applied this test to other barriers. For example, during the epidemic it was suggested that the M6 Motorway, running north-south through Cumbria – and therefore through the centre of the Cumbrian epidemic – may have acted as a barrier to FMDV transmission between farms adjacent to it (Fig. 3, middle inset). Indeed, it is illegal for people, livestock and vehicles to directly cross the M6. We may therefore speculate that infection of farms across the M6 was exclusively via road. The network of minor roads that existed before the M6 was built still exist today – they cross the M6 via numerous tunnels and bridges. Thus, roads between farms on either side of the M6 do not show large excursions as observed around the Severn or the Solway Firth. The *p*-value of the test for IPs within 3 km of the M6 is 0.84 (*n = *188); a non-significant result suggesting that a Euclidean-distance based transmission kernel is a sufficient model of transmission between farms on opposite sides of the M6 Motorway. We have also tested medium to large inland rivers (Devon: *p = *0.64, *n = *296, Cumbria: *p = *0.18, *n = *11700, Dumfries and Galloway: *p = *0.43, *n = *1180) and railway lines (Devon: *p > *0.999, *n = *10, Cumbria: *p = *0.056, *n = *4310, Dumfries and Galloway: *p = *0.56, *n = *351). Thus transmission between farms on opposite sides of these barriers is also best modelled by a Euclidean-distance based kernel rather than a shortest route based kernel. The *p*-value for railway lines in Cumbria is close to significant. However, given that the other regions are not significant it is reasonable to assume the same for Cumbria.

## Conclusion

Why does Euclidean distance work so well, given that some transmission was certainly caused by movement of livestock, people and vehicles between farms via the road network? We do not have a definitive answer, although possible explanations include: 1) farms with a common boundary have more potential routes of infection than just a main road, for example tracks and private roads that cross both farms that are not recorded in the Digimap Meridian™ 2 Database; 2) infection via social networks may be a significant confounding factor.

In conclusion, Euclidean distance between infectious and susceptible farms is a better predictor of transmission risk than shortest or quickest routes, except that is where major geographical features intervene; then shortest route is the preferable measure of distance. Thus, mathematical models of the UK 2001 epidemic were justified in using Euclidean distance as a risk factor. However, future models should take into account the many large estuaries around the UK coastline.

In this paper we have developed a statistical test that can detect risk associated with various measures of the spatial relationship between infectious agents over and above that of simple Euclidean distance. Its use on other economically important livestock diseases may help in understanding their spread in potential future outbreaks. This work stresses the importance of analysing parallel geographical and disease outbreak data in order to construct parsimonious models which capture the essence of disease dynamics and control.

## Methods

### Premises data

The data used in this paper were taken from the DEFRA FMD Data archive [9]. Relevant information for the 2,026 mainland IPs were farmhouse coordinates and infection and slaughter dates. Thirteen IPs in this database that were confirmed on serology tests for antibodies to the virus do not have estimated infection dates; we assume that these IPs were infected 10 days before reporting, which is the period suggested by DEFRA in the database. Data for all other livestock holdings in the UK are an amalgam of 2001 census data and DEFRA's list of premises including all IPs and culled premises from the epidemic; in total 185,791 premises. Relevant information for each premises was farmhouse coordinates.

### Road network

The UK road network was taken from the Digimap Meridian™ 2 Database [10]. In this database, road centre-lines are represented as links, and road intersections as nodes. A road link, which connects two nodes, comprises one or more line segments fixed positionally by a series of connected coordinate points. The coordinate system is the National Grid with a resolution of 1 m. The database distinguishes between Motorways, A roads, B roads and minor roads; it does not include private roads, tracks and some minor roads and cul-de-sacs of less than 200 m. We extract from this database the coordinates of all line segments of all road links. We create our own network of nodes and links, where each line segment is a link connected to two nodes. A node contains a list of all other nodes linked to it, and the Euclidean distance to each of these nodes calculated from the line segment coordinates.

**Figure 5:**
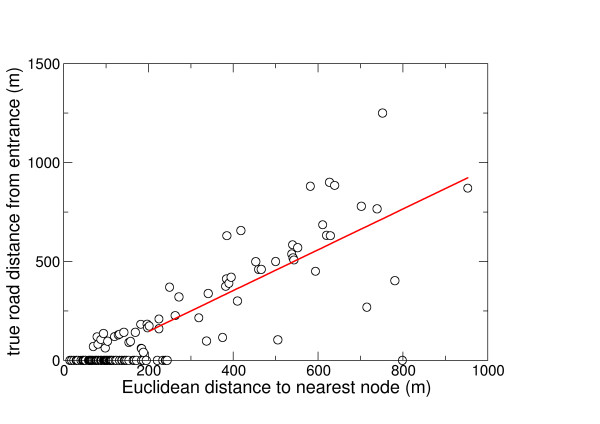
True road distances between farmhouses and their entrances onto a road, taken from Ordnance Survey 1:50000 raster images, against the Euclidean distances between the farmhouses and their nearest nodes in our road network (circles). Linear regression through data with Euclidean distance to nearest node greater than 200 m gives *y *= -60 + 1.03*x*.

Each farmhouse is then assigned to its nearest node in the road network, under the assumption that this node is the closest node to the true farm entrance. The validity of this assumption was checked by hand for 150 randomly chosen premises in Devon, Wales and Cumbria by comparison to Ordnance Survey 1:50000 raster images. Of the 150 premises, 144 (96%) had correctly assigned nodes, the other 4% were assigned a node within 1 km of their correct node. Fig. 5 shows true road distances from the 150 farmhouses to their entrance on a road in our network, as estimated from the raster images, against the Euclidean distance of the farmhouses to their nearest node in the network (in general, the nearest node does not correspond to the position of farm entrance onto a road in our network). Premises can be categorised into two types: those with farmhouses adjacent to a road in the network, which tend to be less than 200 m away from their nearest node; and farmhouses some distance away from a road, which show a linear trend with their distance from their nearest node (these farmhouses are always connected to a road in our network by a road or track not represented in the Digimap Meridian™ 2 Database). We assume that farmhouses less than 200 m away from their nearest node are 0 m away from a road, and that farmhouses greater than 200 m away from their nearest node are -60 + 1.03*x *metres away from a road (from the linear regression shown in Fig. 5), where *x *is the distance to their nearest node. Any redundant nodes are removed from the network to improve computational efficiency. This comprises nodes at dead ends, and nodes that have only two links (in this case, the nodes linking the redundant node are linked together and the distance between them is the sum of the distances between the redundant node and the two linking nodes). A node assigned to a premises is not made redundant.

### Calculating shortest and quickest routes

We calculate the shortest route between all pairs of livestock premises in the UK within 10 km of each other. This is done by analysing 40 × 40 km^2 ^overlapping regions incremented by 10 km horizontally or vertically. This ensures that all farms within 10 km of an IP are linked to an IP by road. Larger regions are computationally infeasible.

The road network in a 40 × 40 km^2 ^region is converted into an *N × N *matrix where *N *is the number of nodes in the region. The matrix is initialised with the road distances between all linked nodes; elements of nodes not linked are given infinite values. The Floyd-Warshall algorithm [11,12] is then applied to this matrix resulting in an *N × N *matrix where the value of each element gives the shortest route between its corresponding pair of nodes. The computational running time of the Floyd algorithm scales as *N*^3^, where *N *varies from approximately 100 to 10,000 depending on the density of roads. When *N *exceeds 10,000 the algorithm's running time exceeds 1 day. The shortest route between any pair of farms is taken as the shortest route between the two assigned nearest nodes to these farms plus the assumed road distance of the farms from the main road. In a very few cases, especially neighbouring farms, the spatial configuration of a pair of farms and their connecting nodes causes the road distance to be less than the Euclidean distance. For these rare cases we assume road distance equal to the Euclidean distance.

To find the quickest route between two farms, distances between two nodes in the network are replaced with journey times. We assume that Motorway and trunk road speeds are 112 kph, A, B and minor road speeds are 72 kph, and farmhouse to road junction speed is 16 kph [13].

### Statistical analysis of distance – based risk

Owing to incomplete or equivocal tracing data, it is not possible to prove conclusively which farm infected which. Therefore we must consider all infectious IPs as possible sources of transmission on the particular day a farm gets infected. However, we can calculate the probabilities of possible transmission events based on known risk factors. We know that risk depends on proximity from an infectious IP (*K*(*d*)) and on the transmissibility () of the infecting farm [5]. Thus, we assume that the probability of an infectious IP *i *infecting a susceptible farm *j *(on the day *t *when *j *was infected) is given by



where (*t*) is the set of all IPs infectious on day *t*. The denominator normalises *p*_*i*,*j *_such that the probability of farm *j *being infected on day *t *is 1. The transmissibilities , are given by [5]

 = *T*_*s*_*N*_*s*,*i*_*+ T*_*c*_*N*_*c*_,_*i*, _    (2)

where *T*_*s *_is the transmissibility of sheep, *T*_*c *_the transmissibility of cattle, *N*_*s*,*i *_the number of sheep and *N*_*c*,*i *_the number of cattle. Only the relationship between *T*_*s *_and *T*_*c *_is required because of the form of Equation 1. We assume that the infectious periods of all IPs begin 3, 4 or 5 days after they become infected and end on the day they are slaughtered [14-16]; the infection and slaughter dates of IPs are taken from the DEFRA FMD Data archive [9].

For a given region, defined in Table 1, only farms in those counties are used in the analysis. For example, for the Cumbria region we assume that only farms in Cumbria can infect Cumbrian farms. Farms in the neighbouring county of Dumfries and Galloway are assumed not to infect Cumbrian farms. Some pre-emptively culled farms may have been infected but never reported. Because it is not possible to say which farms these were or how many of them there were, we cannot include them as IPs in our analysis.

For each IP we find the Euclidean distances and the shortest and the quickest routes between it and all farms it could have infected after 23rd February 2001 and within 10 km (termed possible transmissions), and all farms it could not have infected after 23rd February 2001 and within 10 km (termed non-transmissions). A possible transmission can occur when an IP is infectious on the day another farm was infected (and hence became an IP). A non-transmission between an IP and a farm is defined for three cases: the IP was infectious before the other farm became infected, the IP was infectious before the other farm was pre-emptively culled, and the other farm was never infected or culled.

The mean shortest or quickest route between infectious and susceptible premises in a region is found for possible transmissions (weighted by their probability of occurrence *p*, Equation 1, in which *d*_*i*,*j *_represents Euclidean distance) and for non-transmissions. The difference between these means is recorded. The next step is to compare this difference to a null-distribution. The null hypothesis states that the difference in the means could have arisen by chance. The null-distribution is found as follows. One thousand weighted random samples of possible transmissions are taken from the population of all IP-farm pairs. The sampling is done without replacement. The weighting takes into account the fact that the ratio of possible transmissions to IP-farm pairs varies with Euclidean distance. Therefore, the probability of sampling a possible transmission at a given Euclidean distance is conditioned on this ratio at that distance. If we did not do this, we would preferentially sample IP-farm pairs with longer Euclidean distances within the population because these are more numerous. The unsampled IP-farm pairs make up a random sample of non-transmission pairs. The mean shortest or quickest route of the randomly sampled possible transmissions and non-transmissions are found and their difference calculated. The observed difference in the means is then compared to the null-distribution to obtain a *p*-value.

To test if Euclidean distance is a better predictor of risk than shortest or quickest route, the two variables under consideration are swapped with *d*_*i*,*j *_in Equation 1 representing shortest or quickest route.

### Simulated epidemics

Epidemics were simulated in order to test the power and specificity of the statistical test. The simulations are based on the stochastic simulations done by [5]. Briefly, the infection of susceptible farms are Poisson processes with rates determined by the susceptibility of the susceptible farms, the transmissibility of all infectious farms and a Euclidean-distance or road based transmission kernel. The rates and the Euclidean-distance based kernel are parameterised using the UK 2001 epidemic [5]. If the Euclidean-distance based transmission kernel is *K*_*e*_(*e*) (where *e *is Euclidean distance), and the Euclidean distance-shortest or quickest route density function of IP-farm pairs (e.g., Fig. 2) is *f*(*r*, *e*) (where *r *is shortest or quickest route), then the shortest or quickest route based transmission kernel *K*_*r*_(*r*), is given by



The Euclidean-distance kernel is the black line in Fig. 1. Using farms in Devon for *f*(*r*,*e*), the shortest route kernel is the magenta line and the quickest route kernel is the green line. For the first 30 days of the simulated epidemics, IPs are slaughtered after 3 days of reporting and farms within 1.5 km of an IP are pre-emptively culled after 5 days of reporting. These reduce to 1 and 2 days respectively after the first 30 days. There is no dangerous contact culling. One thousand simulations using the shortest route based transmission kernel were analysed. For an *a *value of 0.05, shortest route was a significantly better predictor of transmission than Euclidean distance for 98% of cases. However, the test for Euclidean distance as a better predictor of transmission was significant in 15% of cases. Conservatively, therefore, our test has a power of about 85%. An additional 1000 simulations using the Euclidean-distance based kernel were analysed. For an *a *value of 0.05, Euclidean distance was a significantly better predictor of transmission than shortest route for > 99.9% of cases. However, the test for shortest route as a better predictor of transmission was significant in just 1% of cases. Conservatively, therefore, our test has a specificity of about 99%.

### Test for best distance – based transmission kernel

The following statistical test was developed to see if transmission between farms on opposite sides of specific transmission barriers is better modelled using a shortest route based transmission kernel or a Euclidean-distance based one. The distribution of infection probabilities (Equation 1) is found for IPs on opposite sides of a barrier first with *d*_*i*,*j*_representing Euclidean distance. The same is then done with *d*_*i*,*j *_representing shortest route. If these two infection-probability distributions are significantly different from each other, this suggests that transmission across the barrier will be modelled differently under the two kernels. Given that transmission did not occur directly over the barrier, this implies that the shortest route based transmission kernel would be the better model. If, however, the distributions are not significantly different from each other, then transmission across the barrier will not be modelled significantly differently under the two kernels; therefore we can assume that a simple Euclidean-distance based transmission kernel will suffice. The Kolmogorov-Smirnov test was used to compare the distributions.

## Authors' contributions

NJS drafted the manuscript. NJS, BTG, MJK, MEJW, SPB conceived the study. NJS, RD, SPB and DS helped with the design of the statistical analysis. DS cleaned and analysed the data and MJT advised the simulation studies. All authors critically assessed the study and read and approved the final manuscript.

## Figures and Tables

**Table 1:** List of regions and the counties used in the analysis.

Region	Counties
Devon	Devon
Cumbria	Cumbria
Dumfries and Galloway	Dumfries and Galloway
Welsh Borders	Powys, Hereford and Worcester, Gloucestershire and Avon
Settle	Lancashire and North Yorkshire

**Table 2:** *p*-values for the test to see if shortest and quickest routes are better predictors of transmission risk than Euclidean distance and vice versa for the various regions and different parameter values. *p*-values below 0.05 are taken to mean a significantly better predictor. The default parameter values are latent period (*l*) equal to 4 days and sheep and cattle transmissibility parameters equal (*T*_*c*_* = T*_*s*_, only relative values are required), *n *is the sum of *p*_*i*,*j *_over all possible transmissions in Equation 1; effectively the number of IPs on their day of infection that are within 10 km of an infectious IP.

		*p*-value				
Region	Parameter	Shortest route better than Eucl. distance	Eucl. distance better than Shortest route	Quickest route better than Eucl. distance	Eucl. distance better than Quickest route	*n*
Devon	*l *= 3	0.55	0.026	0.91	< 10^-3^	107
	*l *= 4	0.68	0.029	0.93	< 10^-3^	104
	*l *= 5	0.61	0.043	0.93	< 10^-3^	103
	*T*_*s*_/*T*_*c *_= 10	0.71	0.027	0.92	< 10^-3^	104
	*T*_*s*_/*T*_*c *_= 0.1	0.65	0.027	0.92	< 10^-3^	104
						
Cumbria	*l *= 3	> 0.999	< 10^-3^	> 0.999	< 10^-3^	572
	*l *= 4	0.999	< 10^-3^	0.999	< 10^-3^	562
	*l *= 5	0.998	< 10^-3^	> 0.999	< 10^-3^	553
	*T*_*s*_/*T*_*c *_= 10	0.998	< 10^-3^	0.999	< 10^-3^	562
	*T*_*s*_/*T*_*c *_= 0.1	0.998	< 10^-3^	0.999	< 10^-3^	565
						
Dumfries and Galloway	*l *= 3	0.87	0.005	0.93	< 10^-3^	82
	*l *= 4	0.82	0.006	0.88	< 10^-3^	78
	*l *= 5	0.82	0.006	0.91	< 10^-3^	76
	*T*_*s*_/*T*_*c *_= 10	0.81	0.005	0.90	< 10^-3^	78
	*T*_*s*_/*T*_*c *_= 0.1	0.85	0.011	0.87	< 10^-3^	78
						
Welsh Borders	*l *= 3	0.87	0.003	0.87	< 10^-3^	97
	*l *= 4	0.88	0.011	0.87	< 10^-3^	93
	*l *= 5	0.92	0.011	0.92	< 10^-3^	88
	*T*_*s*_/*T*_*c *_= 10	0.88	0.012	0.85	0.001	92
	*T*_*s*_/*T*_*c *_= 0.1	0.92	0.008	0.93	< 10^-3^	95
						
Settle	*l *= 3	0.95	0.001	0.96	< 10^-3^	87
	*l *= 4	0.92	< 10^-3^	0.96	< 10^-3^	84
	*l *= 5	0.90	0.001	0.89	< 10^-3^	81
	*T*_*s*_/*T*_*c *_= 10	0.92	< 10^-3^	0.97	< 10^-3^	84
	*T*_*s*_/*T*_*c *_= 0.1	0.91	< 10^-3^	0.94	< 10^-3^	84

**Table 3:** As for Table 2 except that only IPs positively confirmed as infected are considered as IPs. IPs with a negative confirmation are treated as pre-emptive culls. Parameters are *l *= 4 and *T*_*s *_= *T*_*c*_.

	*p*-value				
Region	Shortest route better than Eucl. distance	Eucl. distance better than Shortest route	Quickest route better than Eucl. distance	Eucl. distance better than Quickest route	*n*
Devon	0.55	0.19	0.79	0.008	41
Cumbria	0.93	< 10^-3^	0.998	< 10^-3^	426
D. and G.	0.25	0.055	0.43	0.005	37
Welsh B.	0.13	0.53	0.18	0.33	14
Settle	0.87	< 10^-3^	0.90	< 10^-3^	80
